# Comparison of lower-body neuromuscular performance profiles between 100 m and 400 m Olympic sprinters

**DOI:** 10.3389/fspor.2025.1672028

**Published:** 2025-09-02

**Authors:** Dimitrije Cabarkapa, Amit Batra, Damjana V. Cabarkapa, Andrew C. Fry

**Affiliations:** ^1^ Jayhawk Athletic Performance Laboratory, Wu Tsai Human Performance Alliance, Department of Health, Sport and Exercise Sciences, University of Kansas, Lawrence, KS, United States; ^2^ Faculty of Health Sciences and Physical Education, Kazimierz Wielki University, Bydgoszcz, Poland; ^3^ Faculty of Physical Education and Management in Sport, Singidunum University, Belgrade, Serbia; ^4^ D2 Lab, Novi Sad, Serbia

**Keywords:** vertical jump, sprinting, force plates, asymmetry, female, eccentric, concentric

## Abstract

The purpose of the present study was to examine and compare the lower-body neuromuscular performance characteristics of Olympic-level 100 m and 400 m sprinters. Following a standardized warm-up procedure, fourteen professional female athletes completed three countermovement vertical jumps with no arm swing, while standing on a uni-axial force plate system sampling at 1,000 Hz. Force-time metrics, expressed both in absolute (A) and relative (R) terms, were analyzed across braking (eccentric) and propulsive (concentric) phases of the jumping motion, including inter-limb asymmetry measures. Independent t-tests or Mann–Whitney *U* tests were used to examine between-group statistically significant differences (*p* < 0.05). The results reveal that 100 m sprinters tend to display greater force and power-producing capabilities than their 400 m counterparts. Specifically, average braking power (A: 1,212.3 vs. 1,052.9 W), average propulsive power (A: 2,343.8 vs. 2,026.9 W), peak propulsive power (A: 4,030.1 vs. 3,503.5 W; R: 64.1 vs. 59.4 W·kg^−1^), average propulsive velocity (1.79 vs. 1.69 m·s^−1^), peak propulsive velocity (3.13 vs. 2.93 m·s^−1^), and jump height (47.8 vs. 41.4 cm) were all greater in 100 m than 400 m sprinters. In addition, average and peak braking and propulsive force inter-limb asymmetries remained below 10% in both groups, with no significant differences being observed. Therefore, these findings suggest that 100 m sprinters may benefit from training regimens focused on the development of force and power-producing capabilities, particularly through exercises that enhance the propulsive (concentric) phase of the movement, while 400 m sprinters may benefit from a more integrated training approach, focused on balancing speed development with strength-endurance components.

## 1 Introduction

Sprinting events in track and field, such as 100 m and 400 m races, represent two of the most explosive and physiologically demanding disciplines in this sport. However, they exhibit distinct differences in performance characteristics, including energy system contribution and neuromuscular performance demands (1–6). The 100 m sprint is a predominantly anaerobic event, often referred to as the standard measure of the top speed capabilities of human bipedal locomotion (7). It emphasizes the athlete's ability to rapidly generate and apply the resultant force to propel the body in a forward direction in order to attain peak acceleration and velocity-producing capabilities (7, 8). On the other hand, the 400 m race involves a unique blend of anaerobic and aerobic energy system contributions (1, 4). The 400 m sprinters are required to sustain high-intensity efforts, demanding not only speed and power but also fatigue resistance, metabolic efficiency, and the ability to maintain the optimal biomechanical form under sustained high-speed conditions (1, 2, 6, 9).

Based on the previously published scientific literature, a key physical performance parameter that allows athletes to attain peak acceleration and speed across multiple sprinting distances is lower-body strength (9–14). For example, when examining a cohort of well-trained athletes, Comfort et al. (10) found a strong association between lower-body strength (absolute and relative) and short-distance sprint times, where stronger athletes demonstrated superior sprint performance. Similar findings were obtained by Smirniotou et al. (12), who found that 100 m sprint times were strongly associated with lower-body force and power-producing capabilities in youth track and field athletes. Also, these performance attributes were found to be particularly critical during the early acceleration phase, where high amounts of force in the horizontal direction and greater step frequency are paramount (7, 15). Moreover, in longer sprinting events such as a 400 m race, an athlete's ability to maintain force and power output and optimal stride mechanics under a considerable metabolic strain are some of the key performance determinants in the discipline (16). Thus, the deeper insight into lower-body neuromuscular characteristics of these types of athletes is crucial for developing individualized training regimens and performance monitoring strategies.

Over the years, the countermovement vertical jump (CMJ) has become a widely used method for assessing lower-body force and power-producing capabilities in athletes. Its practicality, simplicity, and non-invasive nature make this test particularly valuable in applied sport settings. In addition, with the growing use of force plate technology in sports science, the CMJ has emerged as one of the most frequently performed tests, providing practitioners with a wide range of objective metrics across different phases of the jumping motion that offer deeper insights into athletes' overall neuromuscular performance capacities (17, 18). As a result, the CMJ has been widely investigated across various team sports (e.g., volleyball, basketball, handball, soccer), with numerous studies supporting its effectiveness in assessing key performance attributes such as force production and fatigue status (17, 19–21). For instance, Cabarkapa et al. (19) have found that the CMJ could detect fatigue-induced changes in neuromuscular performance among professional male basketball players pre-post practice. Additionally, notable sport-specific differences in force-generating capabilities have been reported among professional female athletes in sports such as handball, volleyball, and basketball, with volleyball players generally exhibiting greater explosiveness and superior overall CMJ performance (17). Yet, despite the growing body of literature across team sports, there remains a significant gap in research related to track and field athletes, particularly among top-tier female sprinters (i.e., 100 m and 400 m).

Therefore, to bridge a gap in the scientific literature, the purpose of the present study was to examine and compare the lower-body neuromuscular performance characteristics of female Olympic-level 100 m and 400 m sprinters by using a CMJ test performed on a portable force plate system. Considering the on-field performance demands, it is hypothesized that notable between-group differences will be observed, with 100 m sprinters revealing superior force-time magnitudes when compared to their 400 m counterparts.

## 2 Materials and methods

### 2.1 Participants

A total of fourteen professional track and field female athletes volunteered to participate in the present study, from which eight were 100 m sprinters ($\bar{x}\pm \mathrm{SD}$; age = 25.7 ± 4.6 years; body mass = 62.8 ± 4.8 kg; height = 168.3 ± 4.2 cm) and six 400 m sprinters (age = 25.0 ± 4.9 years; body mass = 59.0 ± 4.8 kg; height = 171.1 ± 4.6 cm). All athletes were an active part of the national team that qualified for the Olympics and were cleared by their respective sports medicine staff for participation in team training activities. The testing procedures performed in the present study were approved by the University's Institutional Review Board and adhered to the Declaration of Helsinki ethical principles.

### 2.2 Procedures

The CMJ testing procedures were conducted as a part of the regular training sessions (10:00–15:00 h). Following a standardized dynamic warm-up protocol (7–10 min) administered by a certified strength and conditioning specialist, each athlete stepped on a uni-axial force plate system (Hawkin Dynamics, Westbrook, ME, USA) and performed three CMJs with no arm swing (i.e., hands on the hips during the entire movement). The sampling rate of the force plate system was 1,000 Hz. The athletes were instructed to push the ground as explosively as possible alongside strong verbal support provided by coaches and teammates (22). Each jump trial was separated by a 15–20 s rest interval. The testing procedures were scheduled 48 h following the last training session to minimize the possible influence of fatigue. The average value across three jump trials was used for performance analysis purposes (19).

The force-time metrics examined in the present study were based on previously published research reports that demonstrated solid levels of validity and reliability, within both braking (eccentric) and propulsive (concentric) phases of the CMJ motion (18, 19, 23–26, 46). The braking phase of the countermovement jump is defined as the period from peak negative velocity of the center of mass until velocity reaches zero, during which the athlete decelerates downward movement and absorbs force eccentrically. The propulsive phase begins immediately thereafter, extending from the zero-velocity point until take-off, and represents the concentric action where upward force production accelerates the body to achieve flight. These metrics were the following: average and peak braking and propulsive force, average and peak braking and propulsive power, braking and propulsive impulse, braking propulsive phase duration, average and peak braking and propulsive velocity, jump height (i.e., the vertical velocity of the system center of mass at the instant of take-off), reactive strength index (RSI)-modified (i.e., jump height divided by contact time), countermovement depth (i.e., peak negative vertical displacement of the system center of mass), and rate of force development (i.e., the average slope of the vertical ground reaction force applied to the system center of mass during the braking phase). To minimize the confounding effect of body mass for between-group comparisons (e.g., peak braking force, average propulsive power), both absolute (N or W) and body mass-normalized values were analyzed (N·kg^−1^ or W·kg^−1^). Moreover, average and peak asymmetries in ground reaction force production during the braking, propulsive, and landing phases of the CMJ motion were obtained. A detailed description of each dependent variable can be found at https://www.hawkindynamics.com/hawkin-metric-database.

### 2.3 Statistical analysis

The Shapiro–Wilk test was used to examine the assumption of normality. If the assumption of normality was met, independent *t*-tests were used to examine between-group differences, variables were reported as mean ± standard deviation $\left(\bar{x}\pm \mathrm{SD}\right)$, and Hedges' *g* was used to depict the effect size magnitude (i.e., *g* < 0.2—small; *g* = 0.5—moderate; *g* > 0.8—large) (27). Conversely, if the assumption of normality was violated, Mann–Whitney *U*-test [median and interquartile range; M (IQR)] were used to examine statistically significant between-group differences the effect sizes were calculated by dividing the Z-statistic with the square root of the sample size (i.e., *r* = Z/√N; *r* < 0.3 small; *r* = 0.3–0.5—moderate; *r* > 0.5—large) (28). The *α* level of *p* < 0.05 was used as a criterion for statistical significance. All statistical analysis procedures were completed in SPSS (Version 28.0; Chicago, IL, USA).

## 3 Results

The descriptive statistics (means and standard deviations or medians and interquartile ranges) for each force-time metric and asymmetry measures examined in this study are presented in Table 1. Between-group statistically significant differences were found in average braking power, average propulsive power (absolute), peak propulsive power (absolute and relative), average and peak propulsive velocity, and jump height, with each variable being greater in 100 m sprinters compared to their 400 m counterparts.

**Table 1:** Descriptive data and statistical comparisons in force-time metrics in a countermovement vertical jump between 100 m and 400 m female Olympic sprinters.

Variable	100 m	400 m	*p*-value	ES
Braking phase				
Average braking force—A [N]	1,330.7 ± 102.9	1,220.1 ± 100.2	0.069	1.087
Average braking force—R^#^ [N/kg]	21.6 (1.9)	19.5 (4.8)	0.662	0.138
Peak braking force—A^#^ [N]	1,707.7 (266.7)	1,569.3 (220.8)	0.142	0.414
Peak braking force—R^#^ [N/kg]	27.9 (4.7)	25.6 (6.5)	0.573	0.174
Average braking power—A^#^ [W]	−1,212.3 (199.0)	−1,052.9 (223.9)	**0** **.** **029** *	0.587
Average braking power—R [W/kg]	−19.7 ± 3.4	−17.6 ± 2.7	0.243	0.684
Peak braking power—A [W]	−1,724.8 ± 296.5	−1,451.5 ± 204.7	0.078	1.073
Peak braking power—R [W/kg]	−27.5 ± 4.5	−24.8 ± 4.7	0.299	0.587
Braking impulse—A [Ns]	190.8 ± 28.2	170.9 ± 27.4	0.212	0.714
Braking impulse—R [Ns/kg]	3.05 ± 0.33	2.89 ± 0.35	0.476	0.472
Braking phase duration [s]	0.144 ± 0.022	0.142 ± 0.029	0.871	0.079
Average braking velocity^#^ [m/s]	−0.985 (0.180)	−0.944 (0.150)	0.181	0.379
Peak braking velocity [m/s]	−1.62 ± 0.19	−1.51 ± 0.15	0.245	0.631
Propulsive Phase				
Average propulsive force—A^#^ [N]	1,453.8 (164.2)	1,316.4 (374.9)	0.573	0.173
Average propulsive force—R^#^ [N/kg]	22.1 (2.2)	21.5 (4.9)	0.414	0.241
Peak propulsive force—A^#^ [N]	1,705.5 (243.3)	1,544.7 (302.7)	0.108	0.448
Peak propulsive force—R^#^ [N/kg]	27.9 (5.1)	26.5 (6.0)	0.576	0.170
Average propulsive power—A [W]	2,343.8 ± 213.8	2,026.9 ± 295.2	**0** **.** **019** *	1.262
Average propulsive power—R [W/kg]	37.3 ± 2.8	34.4 ± 4.9	0.089	0.759
Peak propulsive power—A [W]	4,030.1 ± 340.2	3,503.5 ± 420.4	**0** **.** **012** *	1.402
Peak propulsive power—R [W/kg]	64.1 ± 2.0	59.4 ± 5.9	**0** **.** **026** *	1.145
Propulsive impulse—A [Ns]	344.8 ± 45.8	306.9 ± 34.1	0.116	0.917
Propulsive impulse—R [Ns/kg]	5.47 ± 0.45	5.19 ± 0.36	0.250	0.674
Propulsive phase duration [s]	0.244 ± 0.032	0.238 ± 0.043	0.759	0.162
Average propulsive velocity^#^ [m/s]	1.79 (0.15)	1.69 (0.11)	**0** **.** **039** *	0.517
Peak propulsive velocity [m/s]	3.13 ± 0.16	2.93 ± 0.05	**0** **.** **015** *	1.582
Asymmetry				
Average braking force [%]	4.63 ± 3.65	4.86 ± 3.25	0.906	0.066
Peak braking force [%]	3.74 ± 2.77	4.22 ± 3.01	0.764	0.167
Average propulsive force [%]	3.34 ± 1.31	1.80 ± 1.59	0.071	1.074
Peak propulsive force [%]	3.45 ± 2.85	3.40 ± 2.39	0.976	0.018
Average landing force [%]	7.74 ± 6.59	13.16 ± 10.97	0.270	0.624
Peak landing force [%]	5.05 ± 3.67	5.56 ± 4.50	0.907	0.126
Other				
Jump height [m]	0.478 ± 0.053	0.414 ± 0.020	**0** **.** **016** *	1.506
RFD [N/s]	8,737.3 ± 3,052.7	7,484.3 ± 3,006.5	0.459	0.413
CMJ depth [m]	−0.365 ± 0.087	−0.321 ± 0.060	0.305	0.572
RSI-modified [ratio]	0.667 ± 0.049	0.619 ± 0.119	0.332	0.561

# Variable that violated the assumption of normality.

* Statistically significant between-group difference (*p* < 0.05).

ES, effect size; A, absolute; R, relative; CMJ, counter movement vertical jump; RFD, rate of force development; RSI, reactive strength index.

While no other force-time metric reached the level of statistical significance (*p* < 0.05), it should be noted that the effect size of between-group differences in peak braking power (absolute), average braking force (absolute), average propulsive power (relative), and propulsive impulse (absolute and relative) were all moderate to large in magnitude (*g* = 0.676–1.087), and followed the aforementioned trend of superior lower-body neuromuscular performance characteristics observed in 100 m than 400 m sprinters. In addition, no statistically significant between-group differences were found in age (*p* = 0.743), body mass (*p* = 0.071), and height (*p* = 0.336).

## 4 Discussion

To the best of our knowledge, this is the first study focused on examining differences in lower-body neuromuscular performance characteristics between 100 m and 400 m Olympic-caliber female sprinters. As hypothesized, the results reveal that 100 m sprinters tend to display greater force and power-producing capabilities than their 400 m counterparts. Specifically, average braking and propulsive power (absolute), peak propulsive power (absolute and relative), average and peak propulsive velocity, and jump height were all considerably greater in 100 m than 400 m sprinters. Additionally, despite not reaching the level of statistical significance, the between-group effect size differences for many other force-time metrics were moderate to large in magnitude, such as average braking force (absolute), peak braking power (absolute and relative), peak braking velocity, braking impulse (absolute), propulsive impulse (absolute and relative), and RSI-modified. Thus, further suggesting superior lower-body neuromuscular performance capacities among the cohort of 100 m than 400 m sprinters.

Based on previously published scientific literature, a considerable difference in physiological performance demands exists between these two disciplines, despite both being classified as “short-lasting” track and field events (16, 29). For example, high force and power-producing activities lasting 10–15 s (100 m) heavily rely on anaerobic metabolism as a primary energy source (88%–94% anaerobic/6%–12% aerobic), while longer events that last 45–60 s (400 m) require considerably greater aerobic energy metabolism contribution (55%–63% anaerobic/37%–45% aerobic) (4, 16, 30, 47). Another important factor that needs to be considered is muscle fiber composition (29, 31, 32). Specifically, Costill et al. (33) have found that middle-distance runners (800 m) had a considerably larger percentage of slow-twitch fibers (Type I) than age-matched short-distance sprinters (100 m) and that the athletes' preference for speed or endurance events was in part a matter of their genetic endowment. Similarly, the larger percentage of fast-twitch fibers (Type IIa and IIx) was found to be advantageous in track and field disciplines that require greater reliance on anaerobic energy contribution (32). Thus, based on the aforementioned research reports, we can assume that these physiological adaptations may have predisposed 100 m sprinters to attain superior performance in explosive activities such as CMJ examined in the present study, where athletes are required to rapidly generate as much force as possible within a short period of time (34, 35). Although further research is warranted on this topic, these observations further align with the findings obtained by Fry et al. (36), who found a strong positive association between CMJ power-producing capabilities and Type II muscle fiber characteristics when studying a cohort of Olympic-level weightlifters.

Another consideration when interpreting the findings of the present study is the actual magnitude of CMJ force-time metrics. For example, when compared to a recently published research report by Loturco et al. (37) that studied a cohort of female athletes of similar caliber (e.g., World Championship, Pan-American, Olympic Games), considerably greater peak force and power (absolute) were observed in the present study (1,342.0 vs. 1,705.5 N and 2,891.0 vs. 4,030.1 W, respectively). This discrepancy may be primarily attributed to the differences in body mass between the participants (62.8 vs. 55.4 kg), as when expressed in relative terms, the observed discrepancies in the findings were less pronounced. Also, both studies reported similar vertical jump heights (44.6 vs. 40.8 cm) (37). In addition, higher levels of force and power-producing capabilities have been shown to be of critical importance for successful short-distance sprint performance in order to overcome the inertia of body mass (10). Specifically, maximal lower-body strength (absolute and relative) revealed moderate to strong correlations with both 5 m and 20 m sprint times (10). Besides the previously mentioned research report, this relationship had been well-examined in the scientific literature, where stronger and more powerful athletes were likely to attain better short-distance sprint performance (34, 38–40). When taking into account the on-field performance demands, the 100 m sprinters are required to rapidly produce force within a short time frame, while 400 m sprinters require a mix of speed and endurance capacities (41). Thus, to adequately address the individual athlete's performance needs, the training regimens need to be adjusted accordingly, which ultimately translates to superior CMJ performance capabilities of athletes where high force and power-producing capabilities are imperative (i.e., 100 m sprinters).

Another interesting observation in the present study pertains to the lower-body asymmetry measures. The majority of force-related asymmetries within braking and propulsive phases of the CMJ motion were small in magnitude (<10%). Also, no statistically significant differences were observed in the asymmetry metrics between 100 m and 400 m Olympic-level sprinters examined in this investigation. While inter-limb asymmetries are common in team sports such as basketball, volleyball, and soccer, there is no firm evidence to suggest that they expose athletes to an increased risk of injury (42). However, a couple of studies reported a negative impact of lower-body asymmetries on sprint performance measures, including diminished acceleration and change of direction capabilities (43–45). In the game of basketball, athletes are constantly performing unilateral actions (e.g., shooting layup, one-leg landings), and one limb may be more dominant than the other. On the other hand, sprinting is a cyclic, repetitive, and bilateral movement, where each leg should contribute similarly to optimize acceleration and maintain maximal speed over a certain period of time (36). So, we may assume that these liter-limb asymmetries might be more detrimental to the track and field athletes performance (e.g., 100 m and 400 m sprinters), as small differences in force production could slow them down or make them mechanically inefficient, especially when taking into account that every millisecond may discern an Olympic winner from the rest of the competitors. Thus, considering that these are top-tier athletes, the absence of large inter-limb asymmetries (<10%) might be an indicator of their readiness to compete and adequately developed training regimens to optimize their on-field performance (43), both for athletes participating in 100 m and 400 m disciplines.

While offering a deeper insight into phase-specific differences in lower-body neuromuscular performance characteristics of top-tier 100 m and 400 m sprinters, this study is not without limitations. The athlete sample is homogeneous as it does not include athletes competing on other levels of play (e.g., collegiate, amateur) as well as other middle-distance and long-distance track and field disciplines (e.g., 800 m, 1,500 m, 3,000 m) or longer. Also, the findings might be sex-specific, as the cohort of participants examined in this investigation included only female athletes. However, while the sample size could have been larger, it does represent a unique examination of a very limited number of athletes who were capable of reaching an Olympic-level of competition. Lastly, performing additional tests such as a squat jump, drop jump, or lateral jumps (e.g., unilateral and bilateral) could have been beneficial to obtain a deeper insight into the force-time profile and warrants further research.

## 5 Practical applications

The findings of the present study may offer actionable insights for coaches and strength and conditioning practitioners aiming to optimize performance in sprint athletes. Specifically, the distinct neuromuscular profiles observed between 100 m and 400 m sprinters suggest the need for event-specific training approaches. For example, 100 m sprinters may benefit from training regimens focused on the development of force and power-producing capabilities, particularly through exercises that enhance the propulsive (concentric) phase of the movement (e.g., clean pull, snatch pull, mid-thigh power clean). In contrast, 400 m sprinters may benefit from a more integrated training approach, focused on balancing speed development with strength-endurance components (e.g., sled pushes, uphill sprints, cluster set lifting). In addition, these findings reinforce the use of force plates for CMJ testing as a non-invasive and time-efficient tool for performance monitoring, enabling sports practitioners to design individualized training programs and track neuromuscular adaptations over time.

## Data Availability

The datasets presented in this article are not readily available because of Institutional Review Board restrictions. Requests to access the datasets should be directed to dcabarkapa@ku.edu.
